# The eHealth usage during COVID-19 pandemic 2020 year–Case of Poland

**DOI:** 10.1371/journal.pone.0290502

**Published:** 2023-09-01

**Authors:** Maciej Jankowiak, Justyna Rój

**Affiliations:** 1 Department of Organisation and Healthcare Management, Poznań University of Medical Sciences, Poznań, Poland; 2 Department of Operational Research and Mathematical Economics, The Poznań University of Economics and Business, Poznań, Poland; Silesian University of Technology, POLAND

## Abstract

According to the DESI 2022 digital economy and society ranking, Poland still ranks in one of the last position. Although, in digitising healthcare Poland has made significant progress over the last five years, some inequities in the usage of eHealth have been recognised. This has become an especially important topic after the COVID-19 pandemic. Suddenly, eHealth innovations were much needed to maintain the accessibility of healthcare. Thus, the aim of this study was to explore determinants of eHealth usage by Poles and identify existing potential barriers. Data was collected from the databases of Statistic Poland and statistical methods were employed in this research. The results showed that five variables such as Internet access, Internet use, Internet skills and average monthly disposable income per capita in PLN, along with the number of practicing physicians per capita were important determinants explaining eHealth usage by the analysed Poles between the age of 16–74. The findings showed to increase the usage of eHealth, health policy makers should ensure that Poles acquire and improve Internet skills. Based on results of the research an extended model of eHealth development in Poland, consisting of a central governmental institution and local facilities coordinating remote electronic services, collecting statistical data and providing educational campaigns, was proposed as well.

## Introduction

In recent years, a significant increase in the use of eHealth has been observed, to which restrictions on in-person practice associated with the Coronavirus Disease 2019 (COVID-19) pandemic have also contributed [1]. According to the World Health Organization eHealth means “the cost-effective and secure use of information and communications technologies in support of health and health-related fields, including health-care services, health surveillance, health literature, and health education, knowledge and research” [2, 3]. Broadly speaking eHealth is “health services and information delivered or enhanced through the Internet and related technologies” [4, 5].

The use of eHealth is increasingly recognised as a critical quality driver, as well as a tool to improve equity in healthcare [6–8]. COVID-19 especially highlighted the crucial role of eHealth in offering immediate support in times of need, ensuring safety of communication and delivery of healthcare services during the pandemic, as well as achieving positive health outcomes [9–14]. Remote services of eHealth enabled one to decrease patients’ contagious threats related to direct contact with the healthcare system. Patients using eHealth services were released from the annoying obligation to use COVID-19 protection (like facial masks, body temperature measurements or COVID-19 testing). Additionally, the exploitation of eHealth procedures improved the comfort and effectiveness of medical professionals work due to the possibility of resigning from inconvenient personal protective measures. The risk of COVID-19 transmission among the medical workforce was also minimised, which decreased absence due to sickness. In this time of pandemic, eHealth has shown its value. These kinds of innovations were much needed to maintain the accessibility of healthcare during this period. As a result, medical institutions have intensively integrated eHealth into traditional face-to-face counselling [15].

On the other hand, widespread implementation of eHealth technologies has also raised concerns that it could unintentionally perpetuate healthcare disparities for vulnerable and under-resourced groups [16]. Evidence has begun to emerge that those people who already experience disadvantages and poor health outcomes as well as those individuals with lower socio-economic status are more likely to face eHealth exclusion [17, 18]. Lack of the appropriate skills and / or access to digital tools can increase the disparities in eHealth usage and thus health outcomes [19].

Therefore, it is important to reduce these disparities and avoid further disadvantages [20] especially as equity is one of the important values of the healthcare systems [21]. Equity in health has widely been defined as “the absence of socially unjust or unfair health disparities”. This means the absence of systematic disparities in health between social groups who have different levels of positions in a social hierarchy [22]. Inequity applies to such differences in health that are unnecessary, unfair, and unjust and could be avoided [2], while everyone should have fair opportunity to achieve their full health potential regardless of social, demographic, economic, or geographic status [23]. Thus the rapid increase in the use of eHealth, justifies a renewed focus on health equity [15, 21, 24–31].

For this reason, it is essential to know the socio-economic characteristics of patients and potential patients who are not aware or not able to use eHealth and what barriers these patients face [32]. The usage of eHealth in different countries differs by socio-economic factors [33–39] and such differences in eHealth usage can be observed in Poland [3, 21, 40–45].

The Republic of Poland is a one of the countries within the European Union located in Central and Eastern Europe. In the DESI 2022 digital economy and society ranking, Poland was ranked 24th among the 27 European Union Member States [46] even though in digitising healthcare Poland has made significant progress over the last five years [40]. Up to now, it was found that a lower level of public eHealth services usage was associated with living in rural areas or small cities. Moreover people with higher education are more likely to use these services [40]. However this research covered only a population of 1,092 adult Poles and considered ten socio-economic variables. Earlier, Płaciszewski K, et al. (2022) analysied only nine different socio-economic factors and their relation with the Internet usage for health purposes. They found that being female, having higher education and living in cities with 100,000 to 499,999 residents were the most important factors associated with Internet usage for health purposes [45].

Furlepa et al. (2022) focused only on teleconsultations in primary care in the public sector, and they found that in 2021, there was an apparent increase in the use of medical teleconsultations with age, which is most likely related to the increasing number of diseases occurring with individual age. This could also be the result of an effective educational campaign addressed to seniors, who were asked to limit their activity related to leaving home and interpersonal contacts [44].

Another study examined Internet usage only for the purpose of searching for health information and covering only 1,000 adults Poles and found a connection with the type of living area, level of education and the level of professional activity. Thus, Poles who lived in urban areas, had a high level of education, and were professionally active used more Internet to search for medical information [42], whereas, the use of mobile apps and wearables by a sample of 1070 adult inhabitants of Poland were significantly associated with different socio-economic and lifestyle factors such as younger age, healthy diet, regular physical activity and participation in organised sports activities [43].

Buliński and Błachnio (2017) focused only on telemedical services and found that their usage is still low for the ageing generation and is mainly limited to younger individuals [41]. Such significant age-related barriers of eHealth usage were also observed in the research of [3]. Then, [21] presented the existence of inequities in distribution of only digital determinants of eHealth usage in Poland.

The above studies differ in the size of the population covered by the study–most involve a certain research sample. In addition, they apply to different or various forms of eHealth or diverse scopes of eHealth, as well as socio-economic variables therefore, knowledge about the determinants of eHealth usage by the population in Poland is still scarce. Therefore, to fill in the gap in the area of determinants of eHealth usage, this paper would focus on the more broadly defined eHealth and a wider range of determinants while also covering the entire population of Poles. Based on this, we can then propose a wider range of actions that could be taken to increase the use of eHealth. These elements will constitute the novelty of this research.

Thus, the aim of this study was to explore the determinants of eHealth usage by Poles and identifying existing potential barriers. Thus, this research investigated factors that influence the usage of eHealth by Poles between the age of 16–74. In this way, this study creates a framework and explores determinants of eHealth use by people in Poland. By doing so, this study contributes to the research area of eHealth and would improve the understanding of the factors, which are associated with eHealth usage.

The obtained results may be beneficial for such parties as government, policymakers, healthcare providers and the healthcare device industry as they can support the health policy during development and then implement appropriate eHealth services, as well as education orientation.

This study is structured as follows: Section 1 contains introduction with the theory background and the aim of the study, then data and methods used in this article are described in Section 2; the results are shown in Section 3 and theoretical and practical implications are presented in Section 4; the conclusions of this article were drawn in Section 5.

## Materials and methods

Data was derived from the Statistics Poland database for the year 2020 [47, 48] and „Health and healthcare in 2020” [49]. The underlying data used for the further variable descriptive statistics calculation and the analysis of correlation were presented in S1 Appendix. The range of research covers whole Poland. Variables were collected according to a territorial division of Poland into sixteen units (called regions or voivodeships). In terms of Nomenclature of Territorial Units for Statistics (NUTS) Polish regions represent a level of NUTS 2. Because Poland is divided into sixteen regions each variable consists of sixteen values. The range of variables was defined based on the literature [19, 33–36, 50–54] and then determined by availability of data [47–49].

Using of eHealth services was described by four variables, according to the statistical definitions [47]:

- Information searching: people in age 16–74 searching the Internet for medical information on their health or health of their relatives as a percentage of a total population,
- Visit arranging: people in age 16–74 arranging medical visits via the website or an application as a percentage of a total population,
- Documentation access: people in age 16–74 having an access to a medical documentation via the website as a percentage of a total population,
- Web medical services: people in age 16–74 using services available through the website or an application instead of visiting a doctor or a hospital as a percentage of a total population.

The data relating to a socio-economic profile of the Polish population contain of below listed variables:

- INT—Access: percentage of households (with people aged 16–74) with the Internet access,
- INT—Use: percentage of people aged 16–74 who regularly (at least once a week) use the Internet,
- INT—Skills: percentage of people aged 16–74 who have at least basic skills at the Internet use,
- HD: people who completed university education as a percentage of a total population,
- AVIN: average monthly disposable income per capita in PLN (PLN—Polish currency),
- OLD: number of people in age over 64 for every one person in age 15–64 (as an estimator of an ages structure of a population),
- CIT: people living in cities as a percentage of a total population,
- DOC: practising physicians per capita (here used as an estimator of accessibility to healthcare [55]).

Descriptive statistics of the variables used in analysis of correlation are presented in Table 1.

**Table 1:** Descriptive statistics of variables used in analysis of correlation.

Variable	Mean	Median	Minimum	Maximum	SD	CV
**Information searching**	0.312	0.314	0.208	0,442	0.0553	17.73
**Visit arranging**	0.073	0.071	0.028	0.126	0.0265	36.16
**Documentation access**	0.038	0.040	0.007	0.070	0.0176	46.00
**Web medical services**	0.066	0.063	0.038	0.105	0.0223	33.53
**INT-Access**	0.916	0.921	0.865	0.953	0.0242	2.64
**INT-Use**	0.805	0.793	0.743	0.865	0.0384	4.77
**INT-Skills**	0.480	0.479	0.390	0.626	0.0555	11.56
**HD**	0.007	0.006	0.003	0.011	0.0022	32.23
**AVIN**	1869.6	1867.0	1588.6	2241.0	160.831	8.60
**OLD**	0.281	0.281	0.255	0.318	0.0190	6.75
**CIT**	0.583	0.600	0.410	0.760	0.0967	16.60
**DOC**	0.006	0.005	0.004	0.008	0.0012	21.21

Source: Authors calculations

Abbreviations: SD–standard deviation; CV—coefficient of variation [%]

Coefficient of variation (CV) was employed in assessment of variables variation. CV was calculated with the use of the following formula:

CV[%]=100·(SD/Meanvalue) (1)

where: SD–standard deviation.

In respect of using of eHealth services, searching for medical information on health was the most common. In analysed period about 31% of population employed eHealth service to looking for that kind of information. Remaining three types of eHealth services (arranging of medical visits, access to medical documentation and using web medical services instead of visiting a doctor or a hospital) were much less popular, exploited by about 4–7% of population. eHealth usage variation between regions was at a moderate level, CV of different services utilization was from about 18% to 46%.

Households access to Internet facilities was very good (about 91% of households had the Internet access) and equal (CV was about 2.6%). Usage of the Internet was very good (about 80% of the population aged 16–74 regularly used Internet in all purposes) and almost even (CV was about 4.8%) as well. On the contrary level of Internet skills was moderate (only about 48% of the population aged 16–74 had at least basic skills at the Internet use) and more diversified (CV was about 11.6%).

People who have completed university degree represented only 0.7% of the study population and diversity between regions was moderate (CV was about 32%). Average monthly disposable income per capita in PLN was about 1,870 (almost 430 EURO) at a low diversity level (CV was 8.6%). In examined population for every one person in age 15–64 was almost 0.3 person in age over 64 and CV was low (about 6.8%). Approximately 58% of population were living in cities (CV was moderate– 16%). For one thousand inhabitants was average six practising physicians with moderate differences between regions (CV was about 21%).

Direction and strength of linear correlations between pairs consisting of socio-economic variables and variables describing usage of particular eHealth forms were estimated using the Pearson correlation coefficient (r) calculated as follows:

r=COV(x,y)/SD(x)·SD(y) (2)

where: COV(x,y)–covariance between two variables x and y; SD(x)–standard deviation of variable x; SD(y)–standard deviation of variable y.

A correlation was rated as strong if r was 0.5 and more, moderate if it was between 0.3 and 0.5, and weak if it was below 0.3. Significance of correlation was examined with the use of Student’s t-statistics. Declared level of significance was 0.05. A correlation was recognized as statistically significant if p-value was 0.05 or less.

All analyses were performed using STATISTICA software (TIBCO Software Inc. 2017, Statistica data analysis software system, version 13, http://statistica.io).

## Results

The results of calculation of the Pearson correlation coefficient (r) and p-value are presented in Table 2. Correlations between three forms of the eHealth usage (visit arranging, documentation access, use of web medical services) and majority of socio-economic variables (INT-Access, INT-Use, INT-Skills, AVIN, DOC, CIT and mostly HD) were strong and statistically significant. For this strong significant correlations r was about 0.5 and more, p-value was no more than 0.05. In the case of arranging medical visits via the website or an application the strongest correlations (r above 0.6) were identified for INT-Use and INT-Access. Regarding to an access to a medical documentation via the website the most correlated socio-economic variables were the number of practicing physicians (DOC), INT-Skills and disposable income (AVIN). Use of services available through the website or an application instead of visiting a doctor or a hospital was the most strongly correlated with INT-Skills and INT-Access.

**Table 2:** Correlations between socio-economic determinants and use of particular eHealth forms.

Socio-economic variables	Pearson correlation coefficient, (p-value)			
	Information searching	Visit arranging	Documentation access	Web medical services
**INT-Access**	0.064 (0.815)	0.621 (0.010)	0.494 (0.052)	0.610 (0.012)
**INT-Use**	0.202 (0.453)	0.650 (0.006)	0.498 (0.050)	0.591 (0.016)
**INT-Skills**	0.308 (0.245)	0.572 (0,021)	0.647 (0.007)	0.691 (0.003)
**HD**	0.097 (0.722)	0.515 (0.041)	0.537 (0.032)	0.480 (0.060)
**AVIN**	0.364 (0.166)	0.536 (0.032)	0.644 (0.007)	0.559 (0.024)
**OLD**	0.394 (0.131)	0.273 (0.306)	0.389 (0.136)	0.301 (0.258)
**CIT**	0.231 (0.389)	0.498 (0.050)	0.531 (0.034)	0.519 (0.039)
**DOC**	0.491 (0.053)	0.572 (0,021)	0.670 (0.005)	0.570 (0.021)

Source: Authors calculations

Correlations between those above mentioned three forms of eHealth usage and variables describing the Internet infrastructure or skills (INT-Access, INT-Use, INT-Skills) need the special attention. Most of them (except one) were strong and statistically significant. For five of them r was above 0.6 and p-value was about 0.01 or less that indicated very strong and significant correlation. In case of the next three relationships r was between 0.5 and 0.6, and p-value was 0.05 or less that still showed strong and significant correlation. Only one correlation between documentation access and INT-Access was on the border of strong and significant (r about 0.49, p-value slightly more than 0.05). Implication of this observation that there were strong and significant correlations between eHealth usage and variables describing the Internet exploitation will be more broadly talked over in Discussion section.

Correlations between the last form of eHealth usage (searching information on health with the use of the Internet) and any socio-economic variable were not strong. In this case values of r was below 0.5, although a correlation with the number of practicing physicians (DOC) was on the boundary with strong (r was about 0.49). Additionally some other of interdependencies with health information searching were moderate as well (r was between 0.3 and 0.4). There were correlations with Internet skills (INT-Skills), disposable income (AVIN) and the number of older people (OLD). All of these correlations with searching information on health were no statistically significant, in all cases p-values were above 0.05.

Except mentioned above non-significant relationships with searching the Internet for medical information, there was unable to indicate strong and significant correlations between remaining forms of eHealth usage and some socio-economic variables. First of all there were no such strong correlations between all assessed forms of eHealth exploitation and OLD (however some of these interdependencies were moderate). Furthermore a correlation between use of services available through the website or an application instead of visiting a doctor or a hospital and a level of education (HD) was no strong too (but still were moderate). All of listed here moderate correlations were not statistically significant.

## Discussion

This research provides several major findings. The most general finding is that five analysed variables strongly influence the level of eHealth usage in the form of arrangement of visits, access to documentation and web medical services. These variables are: Internet access, Internet use, Internet skills and average monthly disposable income per capita in PLN with the number of practicing physicians per capita (which is used as an estimator of accessibility to healthcare [55]).

Thus, all digital variables (INT-Access, INT-Use, INT-Skills) appeared to be very important concerning the level of eHealth usage. Therefore, to increase the usage of eHealth, health policy makers should ensure that Poles have the appropriate Internet skills as only 48% of Poles aged 16–74 have at least basic skills when it comes to Internet use. In addition, the value of the variation coefficient at a level of 11.56% shows that this level differs between regions in Poland. Generally the above findings are an indication to the government about the importance and need to organise or provide specific programmes and training to improve the digital skills of Poles, as well as for eHealth applications makers (manufacturers of eHealth technologies) so that they consider the preferences of each specific groups of Poles in designing any eHealth technologies especially as Internet skills matter when using web medical services and access to documentation. As such, this might be a potential barrier to using eHealth.

Regarding the other two variables, the level of accessibility to healthcare (assessed using the number of practicing physicians) and the wealth of individuals, it was found that these are also strong determinants of eHealth usage. First, the observed variability of the number of doctors between regions was positively and strongly correlated with differences between these regions in the exploitation of eHealth in the form of arranging visits, use of web medical services and access to medical documentation. This may result from the substantial shortage of medical professionals in the Polish healthcare system. The problem of the low number of practicing physicians in Poland in comparison to other countries was raised in an earlier publication [55, 56]. eHealth is not a tool which should replace physicians, but it is dedicated to make contact easier with medical professionals. eHealth was especially useful during the COVID-19 pandemic due to enabling access to healthcare services without patients having direct contact with therapeutic centres. This remote access was the cause of the dynamic growth of eHealth services during the COVID-19 pandemic period. Nevertheless, in the case of Poland, the strong correlation between eHealth usage and the number of physicians indicates that there was probably a competition effect between eHealth and classical forms of healthcare based on the shortage of medical professionals in Poland. Further development of eHealth facilities without reducing of medical professionals deficiencies is not enough to improve the level of healthcare in the future.

General economic growth, which could be translated into the increasing wealth of people (measured as average monthly disposable income per capita) was also positively and strongly correlated with eHealth usage. Exploitation of eHealth needs expenditures from both health providers and patients. Health providers have to develop a digital and telecommunications infrastructure, and patients have to possess devices and a connection with the Internet to enable access to eHealth services. This needs extra expenditures, and a higher level of citizens’ income improves their capacity to meet the suitable requirements. In other words, increasing disposable income promotes people’s potential preparedness to exploite eHealth services. Digital exclusion based on insufficient private income could especially lead to the inability to use eHealth and exclusion from a significant (and still growing) part of the healthcare system. In this light, people’s poverty should be reduced, among others, to facilitate citizen’s access to healthcare including eHealth services.

In that case, this research has both theoretical and practical implications in the context of eHealth usage in Poland. It contributes to the broad literature on the determinants of eHealth usage by examining the importance of socio-economic variables for eHeath usage in Poland. Overall, the results provide some reasonable explanation for the use of eHealth by Poles between the age of 16–74.

To date, there are some researches [3, 21, 40–45] which apply to the usage of eHealth in Poland, they differ in range and scope. Thus in comparison to them, these findings extend the range of variables strongly affecting the use of eHealth by variables such as: Internet access, Internet skills and average monthly disposable income per capita in PLN with the number of practicing physicians per capita. Therefore, this study provided a broader picture of eHealth usage in Poland. However, due to differences in the organisation of healthcare systems in different European countries, as well as different medical pattern and culture, a direct comparison between countries is difficult.

These findings matter as along with the growing demand for eHealth–they provide knowledge on the roles of the factors influencing its use. It is important as recognising them is also a crucial step toward defining the success or failure of eHealth spreading among Poles. Thus, this research provides valuable information to eHealth technology developers, service providers, as well as policy makers and enables the planning and carrying out of more effective strategies and policies to endorse eHealth usage by Poles.

The importance of Internet skills and use involves a complex approach but also requires long-term thinking and planning to improve the level of these among Poles. On the one hand, it is necessary to develop the Internet skills of Poles and, on the other hand, to design all applications or programmes so that they are characterised by a high level of usability and convenience for each type of user. In terms of the development of Internet skills of Poles, the focus should be on already familiar users—and in this respect, initiatives at the local level could turn out to be more effective, as they would provide opportunities for greater adaptation to actual needs. For example, in case of seniors all the universities of the third age could be used as they can implement lectures/ exercises on the use of different eHealth technologies but also develop the appropriate skills or extend the programme of existing Internet classes. Moreover, Internet skills and use can be developed among Poles by conducting actions similar to the so-called “White Saturdays”, which are organised locally and allow for the conducting of preventive examinations, especially as such actions enjoy the unwavering interest of patients all over the country. Thus, digital education in the field of healthcare can be organised similarly to “White Saturdays” or accompany them. It will ensure better adaptation to the structure of the local community and thus their needs in the field of eHealth. Such actions would also require the participation of designers and creators of such eHealth technologies as they would also have a chance to collect required information to design and develop better user-oriented programs. As the implementation of solutions which are characterised by a high level of usability and user convenience would increase Internet use in the area of healthcare, the engagement of producers of eHealth technologies in such actions would not only be a source of information but a form of marketing as well. In addition, public support for investments or start-up initiatives should be focused on projects that are friendly to the potential patient.

Taking into account the long-term perspective, school education in this area is also important. As part of classes, children should learn how to use Internet in the field of public services, especially in health services, thus acquiring knowledge on the use of practical applications. Having such skills, they would intuitively be able to use other applications in the field of healthcare in the future.

The obtained results indicate a relationship between the use of eHealth and income. The use of eHealth requires access and the possession of devices of appropriate quality, such as smartphones, webcams or wearable devices. First of all, access to them is associated with incurring certain expenses. Thus, the propensity to buy them will depend on the economic status of a given person or household. Hence, support from the state should also be extended to include another digital tools, i.e. the introduction of tax reliefs for the purchase of specific digital tools related to eHealth (with certain time and quantity limits, of course) and not limiting them to fees for using the Internet. As eHealth has the potential to improve the efficiency of healthcare, in particular with regard to the treatment of patients with chronic diseases, which most often appear as people get older, it would also be worthwhile to introduce specific programmes by the state in the first place that will support this group of people in obtaining the appropriate digital tools.

Moreover, the results indicate the importance of Internet access, which is also conditioned by the existing infrastructure. Hence, it is important for the state to continue its activities aimed at developing and ensuring access to broadband connections, and even to intensify them, in order to provide all Poles—including those living in rural areas or less urbanised areas—with appropriate conditions in this regard and enabling the use of eHealth.

In relation to doctors—as the problem of the lack of practicing physicians per capita [56] has already been noticed by the government, activities in the field of medical education, such as increasing admission limits and the universities educating them, should be assessed positively in light of the research carried out. Certainly, the programme of introducing coordinated care should be intensified, which will make it possible to increase the efficiency of doctors’ work and allow them to devote more time to training in new eHealth technologies.

Thus, any form of institutional support for the development eHealth is needed, either by the implementation of appropriate action plans or taking actions to consolidate all strategies which are connected with the digital health developments. This usually means allocating the appropriate amount of resources and capabilities. In Poland, a governmental programme supporting eHealth development was launched in the 2000s. The programme accelerated during the COVID-19 pandemic. One of the core institutions of this programme is the eHealth Centre, which is responsible for delivery of IT solutions for the national healthcare system [57]. The most important IT arrangement is the eHealth (P1) system. This system integrates remote informatics services both for health suppliers (under the form of electronic drug prescriptions, electronic therapeutic and diagnostic referrals, a register of medical incidences and a system of exchange of some electronic patient records) and for patients (like the Internet Patient Accounts enabling patients to have access to their medical documentation via the Internet, a system of remote medical consultations, electronic registration for medical consultations with use of the Internet and a system of online ordering of drug prescriptions) [58]. Except IT services the e-Health Centre collects and delivers some databases on population health and a health system (like incidence of some diseases, number of medical professionals, number and equipment of some healthcare entities, financing of healthcare), but this data does not cover all essential aspects of eHealth delivery and exploitation. The eHealth Centre organises courses and workshops on delivered eHealth services and is addressed mainly to healthcare suppliers and patients’ organisations. Usage of some eHealth services by healthcare suppliers (like e-prescriptions, e-referrals, maintenance of electronic patients records) is obligatory in light of Polish legislation, which greatly increases their exploitation.

This programme could be an example of a model solution of public support for development of eHealth; nevertheless, based on the results of our research, the programme should be extended with two significant aspects. First, our research showed that one of the key factors for the exploitation of eHealth services by patients was their digital skills. If the eHealth programme was expanded by training courses (also in an online format) directed at individual patients it would improve a patient’s skills in using eHealth services and increase the usage of eHealth services by them.

However the e-Health Centre publishes some statistical data on healthcare and eHealth, the range of this data should be significantly extended to facilitate exact analyses of the needs and outcomes of the eHealth system. First, it is necessary to collected more detailed data, both geographically (not only at the level of the country or regions, but also at a level of counties and local communities) and demographically (with a specification of gender, age group, place of living–town or village). Second, more precise data on the use of specific eHealth services is necessary as well. This data should be publicly accessible with the purpose of preparation of analysis eHealth system effectiveness and eventual proposals for its improvement.

This study has certain limitations. First, research was conducted based on the population of Poles between the age of 16–74. Therefore, future research could cover larger populations of Poles even in the age group above 74, as exclusion of the older population from active Internet users could be misleading. In this way, this research could show the statistical office and healthcare decision makers how important the extension of the acquired and collected data is; otherwise, the possibility of conducting detailed research on the usage of eHealth is limited.

Then, this study is limited with regard to the way eHealth is defined according to statistical purpose. Therefore, future research could be extended by analysing each separate technology and form of using the Internet (computer, phone etc), as well as eHealth technologies (e.g., robots) or other health information technologies, which were not included in the research and which are use also by the supply side of healthcare. All the limitations of this study may create opportunities for future academic research on the socio-economic determinants of eHealth usage.

Based on the findings of this research it is possible to propose an extended model of eHealth development in Poland. A core component of the model should be a central governmental institution (similar to now existing the e-Health Centre), which coordinates all central and local actions in the field of eHealth. This core institution except providing unified health informatics solutions, should be responsible for acquiring and processing adequate and detailed statistical data necessary for monitoring and evaluating of a process of eHealth services delivery and usage. This institution is supposed to coordinate educational actions addressed both to patients and healthcare providers and to promote wide using of eHealth as well. The central eHealth institution shall be supported by local facilities, which assist in communication with local societies, implementing of local promotional and educational actions, and statistical data collecting at a local level. Appropriate legislation enabling eHealth implementation and usage should be adopted as well.

## Conclusions

This paper focused on the factors influencing the use of eHealth by Poles between the age of 16–74 during the COVID-19 pandemic. This study explored the determinants of eHealth usage by Poles and identified the existing potential barriers in this area. The results revealed that five variables such as Internet access, Internet use, Internet skills and average monthly disposable income per capita in PLN with the number of practicing physicians per capita were important determinants explaining eHealth usage in the analysed group of Poles. Thus, the findings showed that to increase the usage of eHealth, health policy makers should ensure that Poles have appropriate Internet skills and to develop programmes to increase the digital literacy of the population. Based on these findings, the possibility of extending eHealth usage in Poland also was proposed. The postulated model of eHealth development contains of a central governmental institution supporting by local facilities, which is responsible for coordinating of implementation and usage of eHealth services, collecting detailed statistical data and providing educational or promotional actions. This showed that institutional support is needed at both the central and local level.

Thus, the findings filled the research gap of eHealth usage in Poland as previous research in the area of socio-economic determinants of eHealth usage were conducted in a narrower scope. Moreover, these findings could help practitioners carry out feasible plans to develop and facilitate the usage of different types of eHealth services technologies.

## Supporting information

S1 Appendix The underlying data (from 16 Polish regions) used for the variable descriptive statistics. (DOC)
