# Supplementary material for: The eHealth usage during COVID-19 pandemic 2020 year–Case of Poland
Source: PLoS One. 2023 Sep 1;18(9):e0290502. doi: 10.1371/journal.pone.0290502 (PMC10473534; doi:10.1371/journal.pone.0290502)
Supplement: S1 Appendix — (DOC) [file pone.0290502.s001.doc]

**S1 Appendix. The underlying data (from 16 Polish regions) used for the variable descriptive statistics calculation and the analysis of correlation**

| Region  (Voivodeship) | Total popu-lation^a^ | Informa-tion searching^b^ | Visit arranging^b^ | Documen-tation access^b^ | Web medical services^b^ | INT-Access^b^ | INT-Use^b^ | INT-Skills^b^ | HD^a^ | AVIN^a^ | OLD^a^ | CIT^a^ | DOC^a,c^ |
| --- | --- | --- | --- | --- | --- | --- | --- | --- | --- | --- | --- | --- | --- |
|  | mln | Units of measurements – see the text of the paper | | | | | | | | | | | |
| DOLNOSLASKIE | 2.89 | 0.291 | 0.101 | 0.062 | 0.088 | 0.994 | 0.844 | 0.529 | 0.010 | 2 031.24 | 0.299 | 0,68 | 0,0058 |
| KUJAWSKO-POMORSKIE | 2.06 | 0.268 | 0.076 | 0.023 | 0.100 | 0.934 | 0.841 | 0.520 | 0.006 | 1 845.34 | 0.275 | 0,59 | 0,0055 |
| LUBELSKIE | 2.10 | 0.334 | 0.079 | 0.045 | 0.070 | 0.903 | 0.743 | 0.427 | 0.008 | 1 679.02 | 0.288 | 0,46 | 0,0066 |
| LUBUSKIE | 1.01 | 0.268 | 0.062 | 0.038 | 0.043 | 0.909 | 0.762 | 0.475 | 0.003 | 1 971.47 | 0.274 | 0,65 | 0,0046 |
| LODZKIE | 2.44 | 0.372 | 0.073 | 0.048 | 0.070 | 0.891 | 0.793 | 0.481 | 0.006 | 1 871.83 | 0.318 | 0,62 | 0,0067 |
| MALOPOLSKIE | 3.41 | 0.208 | 0.064 | 0.041 | 0.052 | 0.921 | 0.788 | 0.512 | 0.011 | 1 914.74 | 0.262 | 0,48 | 0,0070 |
| MAZOWIECKIE | 5.43 | 0.442 | 0.126 | 0.070 | 0.105 | 0.953 | 0.578 | 0.626 | 0.010 | 2 240.52 | 0.286 | 0,64 | 0,0081 |
| OPOLSKIE | 0.98 | 0.241 | 0.042 | 0.007 | 0.061 | 0.898 | 0.793 | 0.426 | 0.005 | 1 711.34 | 0.288 | 0,53 | 0,0043 |
| PODKARPACKIE | 2.12 | 0.325 | 0.047 | 0.017 | 0.044 | 0.932 | 0.761 | 0.476 | 0.006 | 1 588.57 | 0.257 | 0,41 | 0,0048 |
| PODLASKIE | 1.17 | 0.327 | 0.028 | 0.026 | 0.061 | 0.883 | 0.782 | 0.489 | 0.007 | 1 948.31 | 0.272 | 0,61 | 0,0054 |
| POMORSKIE | 2.35 | 0.311 | 0.114 | 0.048 | 0.073 | 0.921 | 0.843 | 0.465 | 0.009 | 1 799.12 | 0.265 | 0,63 | 0,0052 |
| SLASKIE | 4.49 | 0.363 | 0.103 | 0.050 | 0.102 | 0.936 | 0.841 | 0.501 | 0.006 | 2 050.36 | 0.301 | 0,76 | 0,0068 |
| SWIETOKRZYSKIE | 1.22 | 0.341 | 0.061 | 0.027 | 0.038 | 0.865 | 0.775 | 0.390 | 0.005 | 1 726.92 | 0.307 | 0,45 | 0,0054 |
| WARMINSKO-MAZURSKIE | 1.42 | 0.316 | 0.053 | 0.015 | 0.042 | 0.902 | 0.809 | 0.408 | 0.005 | 1 884.05 | 0.255 | 0,59 | 0,0041 |
| WIELKOPOLSKIE | 3.50 | 0.291 | 0.069 | 0.039 | 0.064 | 0.937 | 0.782 | 0.474 | 0.008 | 1 789.13 | 0.261 | 0,54 | 0,0038 |
| ZACHODNIO-POMORSKIE | 1.69 | 0.293 | 0.075 | 0.054 | 0.049 | 0.922 | 0.865 | 0.484 | 0.005 | 1 789.13 | 0.289 | 0,68 | 0,0053 |

Source: a - Statistics Poland 2020; available from: [https://stat.gov.pl](https://stat.gov.pl/) [48]; b - Statistics Poland Database. 2019–2020. Wykorzystanie technologii informacyjno-komunikacyjnych w jednostkach administracji publicznej, przedsiębiorstwach i gospodarstwach domowych w 2020 roku; available from: https://stat.gov.pl/obszary-tematyczne/nauka-i-technikaspoleczenstwo-informacyjne/spoleczenstwo-informacyjne/wykorzystanie-technologii-informacyjno-komunikacyjnychw-jednostkach-administracji-publicznej-przedsiebiorstwach-i- gospodarstwach-domowych-w-2020-roku,3,19.html [47]; c - Łysoń P. editor. Health and health care in 2020. Warszawa, Kraków: Statistics Poland. Statistical Office in Kraków. Departament Badań Społecznych. 2021 [49].
